# Clinical Challenges in the Management of Leishmania/HIV Coinfection in a Nonendemic Area: A Case Report

**DOI:** 10.1155/2012/787305

**Published:** 2012-12-17

**Authors:** K. Grabmeier-Pfistershammer, W. Poeppl, P. M. Brunner, K. Rappersberger, A. Rieger

**Affiliations:** ^1^Division of Immunology, Allergy and Infectious Diseases, Department of Dermatology, Medical University of Vienna, 1090 Vienna, Austria; ^2^Department of Infectious Diseases and Tropical Medicine, Medical University of Vienna, 1090 Vienna, Austria; ^3^Department of Dermatology and Venereology, Rudolfstiftung Hospital, 1030 Vienna, Austria

## Abstract

We report on a 37-year-old male HIV-positive patient with generalized cutaneous leishmaniasis undiagnosed for several years. Upon presentation, visceral leishmaniasis was diagnosed in addition to cutaneous manifestation of the disease. Over a period of three years, several different treatment regimens including liposomal amphotericin B, liposomal amphotericin B with miltefosine, liposomal amphotericin B with interferon, and pentamidine combined fluconazole and allopurinol were applied until Leishmania PCR from blood turned negative. This case supports the necessity of multidrug combinational and sequential therapy over a very prolonged period of time in severely immunosuppressed patients infected with Leishmania and highlights the tremendous individual but also economic burden of this disease.

## 1. Introduction

In areas where both HIV and leishmaniasis are endemic, leishmaniasis—although not included in CDC AIDS definition—has gained clinical importance as opportunistic infection in HIV-infected individuals. In the immunocompromised host, this infection poses diagnostic as well as therapeutic challenges since serology is not reliable, the clinical presentation may be unspecific, and therapeutic failure and relapse are common [1]. In the recent years, an increase in leishmaniasis incidence and prevalence has been noticed also in nonendemic countries not only due to increased international travelling, but also by the spread of the sandfly vector, further highlighting the relevance of this disease [2, 3].

## 2. Report

**Figure 1:**
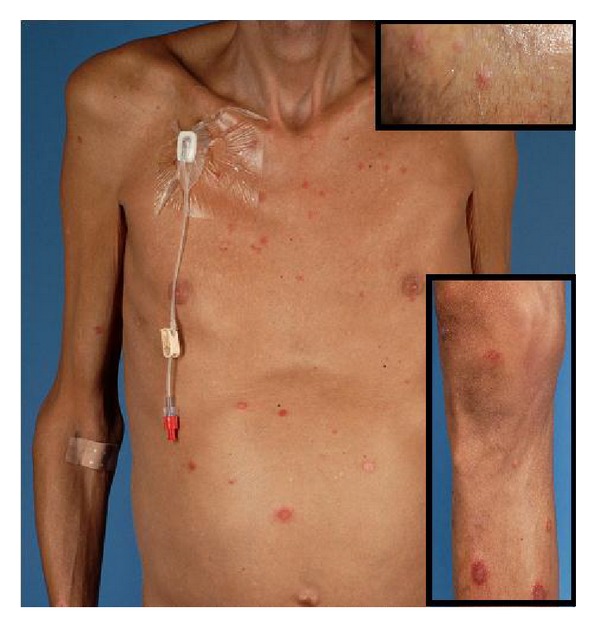
Generalized multiple erythematous, pustular plaques, papules, and nodules preexisting since several years in an HIV-positive patient with cutaneous and visceral L. donovani/infantum complex infection.

**Figure 2:**
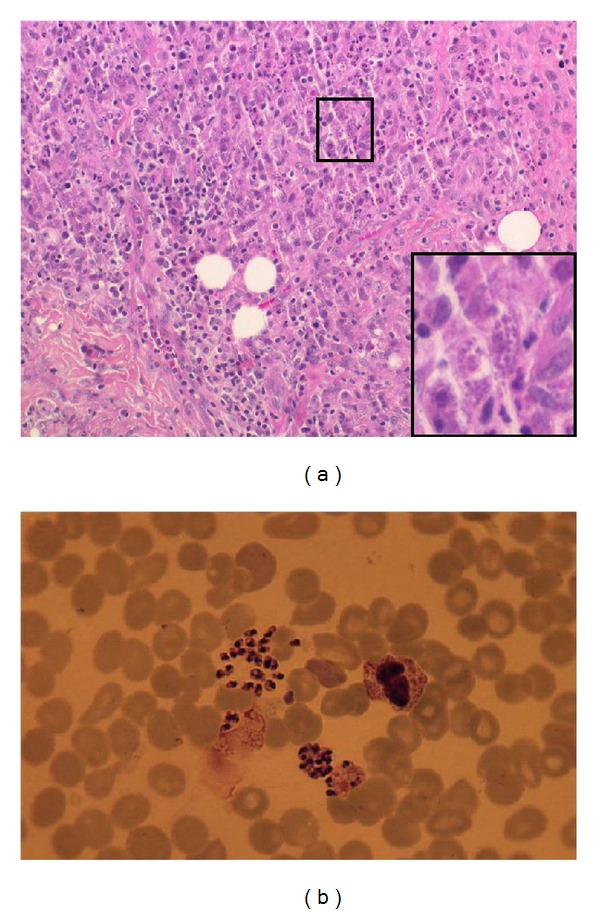
(a) Skin biopsy demonstrating a dense infiltrate of histiocytic cells with Donovan bodies. (b) Donovan bodies in bone marrow aspiration.

In June 2009, a 37-year-old man presented at our out-patient clinics with generalized multiple erythematous, pustular plaques, papules, and nodules preexisting since several years (Figure 1). In addition, in physical and laboratory examination, severe cachexia, pancytopenia, and hepatosplenomegaly were evident. The patient was known to be HIV positive for about 20 years, and in spite of long-term virological suppression, CD4 cells never exceeded 100 cells/*μ*L. A skin biopsy revealed a dense infiltrate of histiocytic cells with Donovan bodies compatible with cutaneous leishmaniasis (Figure 2(a)). Leishmania serology was negative, but PCR from blood and skin samples was positive for L. donovani/infantum complex. In addition to cutaneous leishmaniasis, bone marrow aspiration showed infiltration with Leishmania confirming visceral leishmaniasis (Figure 2(b)). The patient reported yearly holiday trips to Ischia (Gulf of Naples) during prior decades.

Systemic first-line therapy with liposomal amphotericin B (iv, 4 mg/kg days 1–5, day 10, then 5 times in weekly intervals) was started. Since lower response rates to first-line therapy in HIV-infected patients are described, miltefosine (po, 100 mg/day) was added to the standard amphotericin B regimen. Initial response to treatment was adequate, the patient gained weight, the pancytopenia improved, CD4 cell count increased, and Leishmania PCR from blood was negative. HI-viral load was suppressed at any time point. Due to the poor CD4 response and the high relapse rate described in HIV-infected patients, the previous induction regime was followed by maintenance therapy with liposomal amphotericin B (iv, every 3 weeks) and miltefosine (po, 50 mg thrice weekly) planned to be administered until immune recovery (i.e., >350 CD4 cells/*μ*L) has been repeatedly documented. Nevertheless, one year after the treatment initiation, Leishmania PCR from blood turned positive again. Thus, a second induction cycle of liposomal amphotericin and miltefosine (po, 100 mg/day) was started. In spite of clinical response to treatment, Leishmania PCR remained positive over the next six months. Therefore, miltefosine was stopped and replaced by interferon gamma (100 *μ*g sc 3 times/week). Initial tolerance to interferon was good, but after 12 weeks, the patient developed severe leukopenia (1950/*μ*L) and thrombopenia (24000/*μ*L), and interferon gamma administrations had to be stopped. An extensive re-evaluation after 2 years after diagnosis and treatment start revealed Leishmania parasites in blood, bone marrow, gastric mucosa, and the biliary tract. Based on data reporting treatment approaches with pentamidine and the successful use fluconazole and allopurinol in patients with mucocutaneous and visceral leishmaniasis [4, 5], we started a new therapy cycle combining pentamidine (iv, initial dose 4 mg/kg, then 3 mg/kg), fluconazole (po, 200 mg/day), and allopurinol (po, 300 mg/day) for 3 weeks followed by maintenance therapy with allopurinol and intravenous pentamidine every 3 weeks. Therapy with pentamidine was complicated by recurrent hypertensive crisis and two episodes of pulmonary edema requiring intensive care. Nevertheless, this treatment regime led to an instant and impressive recovery of hematopoiesis, a continuous rise of CD4 cells (absolute count and percentage), and last but not least to undetectability of Leishmania in peripheral blood and bone marrow.

## 3. Discussion

Visceral leishmaniasis in HIV-infected patients emerged as a serious opportunistic infection. In endemic areas, up to 30% of HIV-infected patients present with leishmaniasis, and in southern Europe, 70% of visceral leishmaniasis occur in HIV-infected patients [1]. HIV infection increases the risk of developing visceral leishmaniasis, and leishmaniasis can accelerate HIV progression [1, 6]. Furthermore, leishmaniasis in HIV coinfection tends to present with less specific symptoms. Thus in nonendemic areas like in the patient described earlier, diagnosis is often delayed. In addition, commonly used diagnostic practice such as antibody testing is not reliable in coinfected patients, and the diagnosis rather has to rely on direct pathogen detection by molecular methods like PCR and/or histological examination [6, 7]. Therapy is complicated by higher rates of toxicity, relapse rates, and lower cure rate with initial regimen. Although the introduction of combination antiretroviral treatment (CART) has changed the outcome of AIDS-associated leishmaniasis, concordant HIV infection and visceral leishmaniasis still carry high rates of relapse and mortality [8]. CART mainly seems to have a beneficial effect on incidence rates of transition from asymptomatic to symptomatic leishmaniasis and the survival. Its effect on relapse rates, however, is less clear. It has been estimated that up to 70% of coinfected patients end up with a relapse within 24 months [9]. CART might delay but not prevent relapse of leishmaniasis [6]. While a recent study in India reported 95% response rate of a single dose of liposomal amphotericin in HIV non-infected patients, response rates for coinfected patients, even on prolonged treatment regimens, are much lower [10, 11]. The most important prognostic factor for response to therapy and relapse rate is the degree of immunosuppression. A systemic review performed on 18 independent studies could identify a CD4 cell count below 100 cells/mL as well as absence of CD4 increase upon followup as main risk factors for disease recurrence [12]. However, bone marrow infiltration, pancytopenia, and chronic immune activation by Leishmania present major obstacle to immune recovery and long-term remission. Secondary prophylaxis seemed to have an impact on recurrence rates but did not prevent it completely. Furthermore, in contrast to other opportunistic infection, relapse can occur even at CD4 cell counts above 200 cells/mL. It was postulated that despite of increased CD4 cell counts as a result of CART, it takes several months until a cytokine profile favourable for the elimination of Leishmania is provided [12]. So, for safe discontinuation of Leishmania maintenance therapy, it seems useful to take a threshold well above this level for a prolonged time period.

The reasons for failure of maintenance therapy are not completely understood. While the case presented here and others are suggestive for gradual resistance development, a recent study has shown susceptibility of the parasite to amphotericin even in presence of treatment failure [13]. We could not test for resistance in our patient and thus retried amphotericin in a second cycle which in our hands did not lead to elimination of Leishmania.

So in conclusion, this case supports the necessity of multidrug combinational and sequential therapy over a very prolonged period of time in severely immunosuppressed patients infected with Leishmania and highlights the tremendous individual but also economic burden of this disease.
